# Non-ketotic Hyperglycemia Presenting as Focal Status Epilepticus and Subsequent Todd’s Paralysis

**DOI:** 10.7759/cureus.63460

**Published:** 2024-06-29

**Authors:** Kholoud Aljaberi, Nihal Salih, Akhil Narayanan Palat, Sudhir Kumar Palat Chirkkara

**Affiliations:** 1 Neurology, Sheikh Shakhbout Medical City, Abu Dhabi, ARE; 2 College of Medicine, Kasturba Medical College of Mangalore, Mangalore, IND

**Keywords:** todd’s paralysis, diabetes, hyperglycemia, status epilepticus, seizure, epilepsy

## Abstract

Uncontrolled diabetes can result in many neurological and non-neurological complications. It’s common for hypoglycemia to present as a seizure; however, in cases of hyperglycemia, especially in the absence of ketones, seizures are uncommon. Here, we present a case of a 75-year-old female with no prior history of epilepsy disorder presenting as focal status epilepticus complicated by Todd’s paralysis. We are describing the case with a review of the current literature.

## Introduction

Non-ketotic hyperglycemia (NKH) is a complication of diabetes mellitus, characterized by elevated blood glucose levels above 200 mg/dL and high serum osmolality without ketones in urine [1]. This can lead to seizures and involuntary movements, most commonly hemichorea-hemiballism, which is also known as diabetic striatopathy, and those are considered to be rare manifestations of non-ketotic hyperglycemia; however, it carries a good prognosis as the resultant disorders resolve with the correction of the underlying metabolic disturbances. Some may need additional symptomatic management to control the seizures or the abnormal movements; hence, anti-epileptics or neuroleptics may be added if the patient is still symptomatic despite glucose correction [2]. Literature search didn’t reveal any prior similar case reports from UAE.

## Case presentation

**Figure 1:**
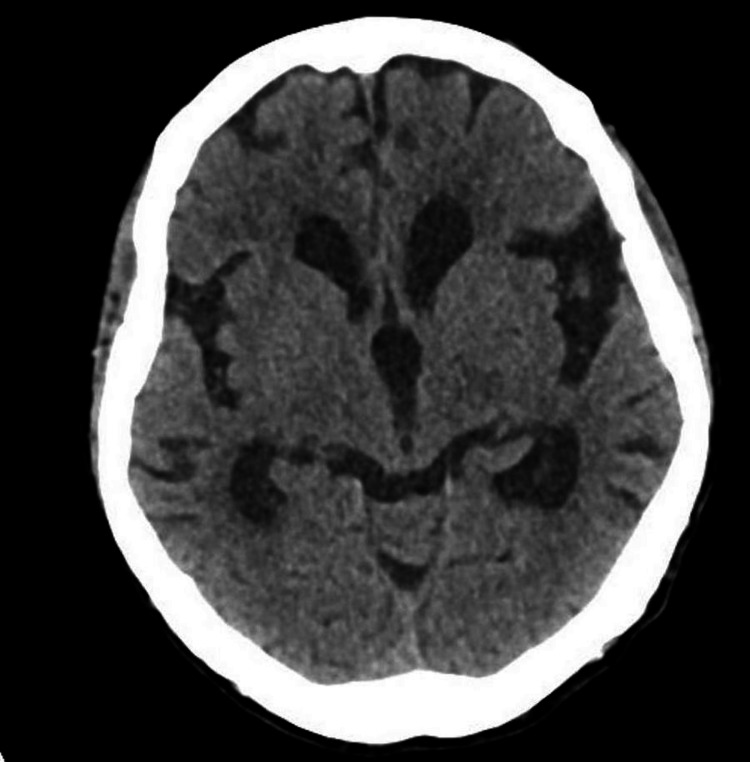
CT head, axial view, no bleeding, no acute changes suggestive of stroke, ASPECT score is 10. ASPECT: Alberta Stroke Program Early CT Score.

**Figure 2:**
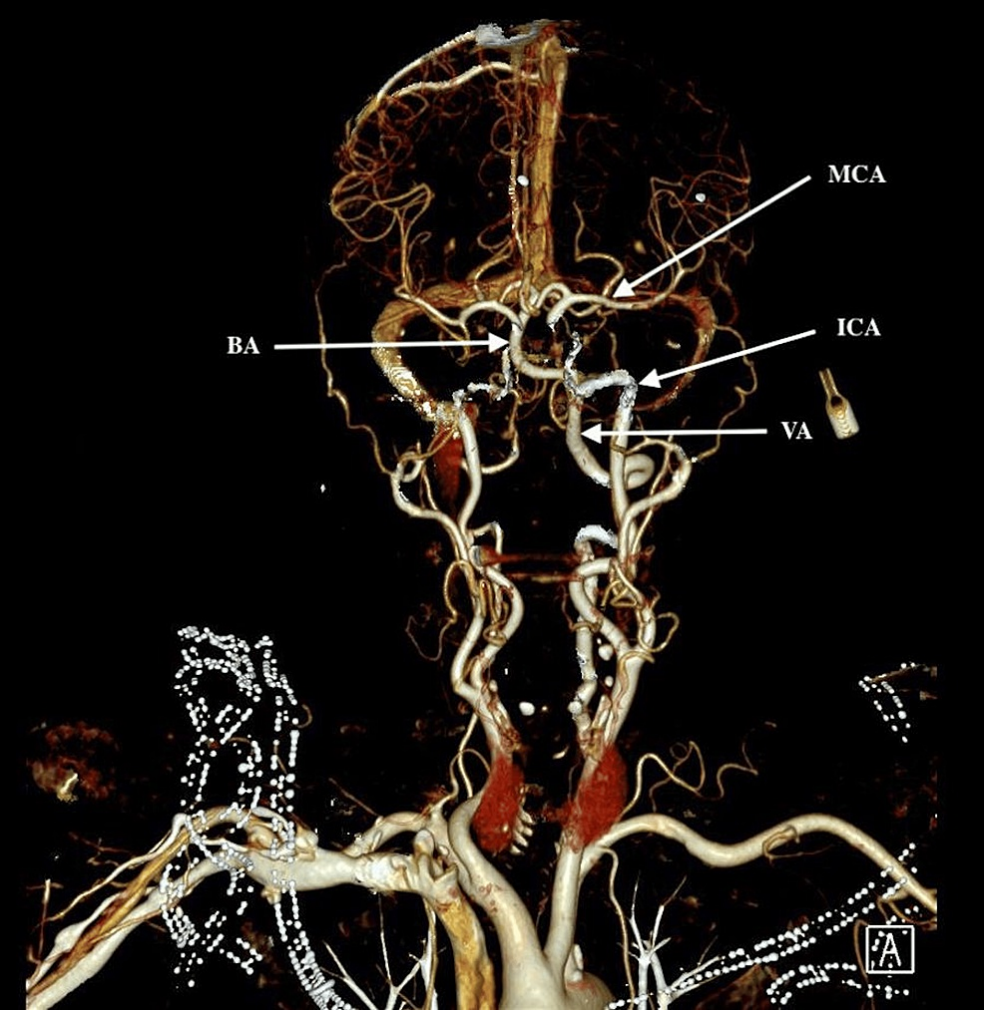
CT angiography, head, and neck major vessels are shown; no large vessel occlusion. BA: basilar artery, MCA: middle cerebral artery, ICA: internal carotid artery, VA: vertebral artery.

A 75-year-old female with type 2 diabetes, peripheral vascular disease, status post-left lower limb amputation, hypertension, and no history of epilepsy. She presented with acute onset of jerky movements affecting her left face and limbs with preserved consciousness of two hours duration. Clinically, she was alert but disoriented. Pulse 98/minute, BP 236/117 mm of Hg, afebrile, saturation of 99% in room air. The focal seizure activity involved the left side of the face, left upper limb, and left lower limb stump. She could obey commands with the right side of the body. The seizure activity could be controlled with IV lorazepam and a loading dose of levetiracetam. Her labs showed elevated blood glucose of 38.7 mmol/L, increased calculated serum osmolality 313 mOsmol/kg, low serum Na-134 mmol/L (136-145 mmol/L) and low phosphate-0.79 mmol/L (0.81-1.45 mmol/L), normal potassium-4.8 mmol/L and magnesium-0.74 mmol/L, and normal lactate of 2.2. CT head (Figure 1) and CT angiography head and neck (Figure 2) were done as part of a code stroke that was normal.

**Figure 3:**
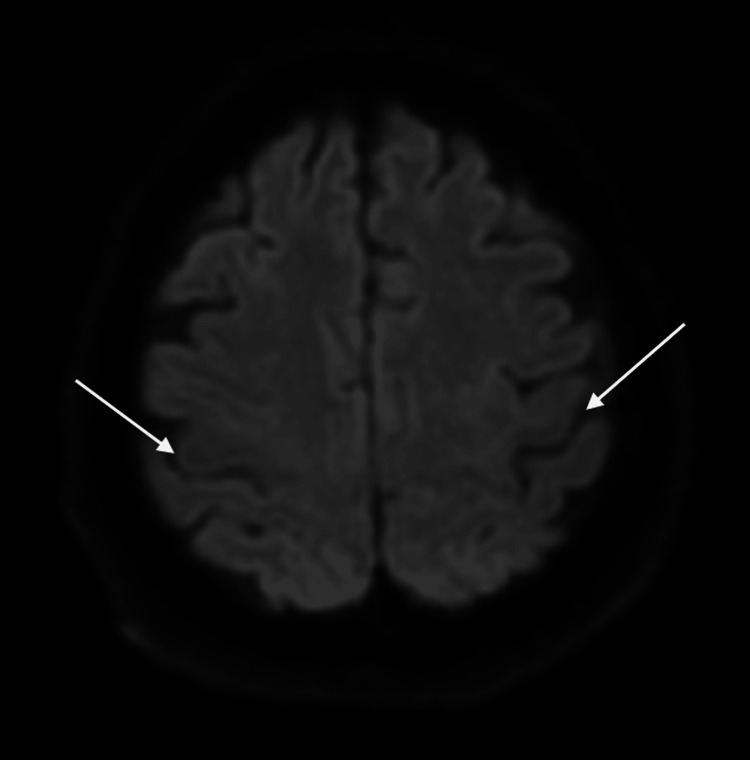
The MRI of the brain shows an axial view and diffusion-weighted imaging (DWI). Arrows pointing at the central sulci; there is no diffusion restriction in the precentral gyri anterior to the central sulci or any other area.

**Figure 4:**
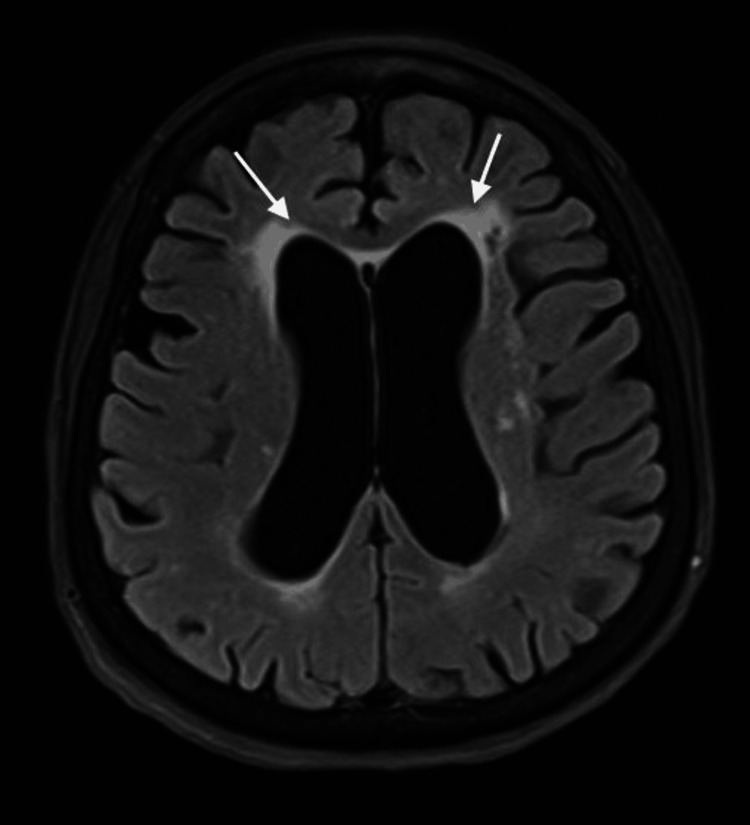
The MRI of the brain shows an axial view and a fluid-attenuated inversion recovery (FLAIR) image. Arrows pointing at periventricular hyperintensities representing small vessel disease.

When she woke up from sedation the next day, the motor power was 0/5 MRC on the left side, with flaccidity and sluggish reflexes. She was conscious of obeying commands. Her blood glucose was trending down; the result of HbA1C came back elevated, as it was 11.2%. A brain MRI stroke protocol (Figures 3, 4) was negative for new vascular insults. The EEG did not reveal any epileptiform discharges. By 48 hours, she started to regain motor power and came back to her baseline by 72 hours. The final diagnosis was focal status epilepticus complicated by Todd’s paralysis precipitated by non-ketotic hyperglycemia.

## Discussion

Uncontrolled diabetes with hyperglycemia can result in a wide spectrum of neurological issues, such as hemiparesis, hemisensory loss, encephalopathy, peripheral neuropathy, autonomic neuropathy, neuropathic osteoarthropathy, focal seizures, and different forms of movement disorders like hemiballismus, chorea and athetosis [3,4].

The first report of focal seizures induced by hyperglycemia was published in 1965 [5]. Uncontrolled hyperglycemia is a major issue in elderly people with diabetes. Seizures are usually associated with non-ketotic hyperglycemia and are mostly encountered in patients over the age of 50 with a longstanding history of diabetes. In some cases, seizures are the presenting symptom in undiagnosed patients with diabetes [6]. 

In a retrospective observational study, non-ketotic hyperglycemia-induced seizures were observed in two different situations: firstly, in the case of pre-existing vascular epilepsy and acute stroke; secondly, seen as a reversible metabolic phenomenon, brain MRI is essential to make this distinction between the two groups [1].

Seizures present differently, motor focal seizures represent about 75-86% of the cases, generalized tonic-clonic seizures are rare, and some patients may present with partial status epilepticus [6].

The exact mechanism by which hyperglycemia affects neurological status is not very clear; however, several theories have been proposed. One is that the Krebs cycle is inhibited by hyperglycemia, leading to gamma-aminobutyric acid (GABA) depletion by increased metabolism, which lowers the threshold of seizure, or by hyperglycemic damage to cerebral vessels, causing transient ischemic disease [7]. Another explanation is increased intracellular osmotic pressure, leading to nerve cell dehydration accompanied by altered enzyme activity, dysfunction of membrane ion pump and the stability of cell depolarization [8].

MRI findings in cases of hyperglycemia-induced seizure are usually localized and characterized by the presence of subcortical T2 hypointensity with low signal on apparent diffusion coefficient (ADC), gyral hyperintensity involving the cortex with leptomeningeal post-contrast enhancement [9]. In a study of three cases, all presented as focal seizures, all MRIs showed subcortical T2 and FLAIR hypointensity with or without cortical T2 hyperintensity. In follow-up imaging, two cases had a complete resolution of the MRI findings, and the other had a significant reduction of the abnormal signals [10]. Other focal lesions may be seen on imaging that are unrelated to hyperglycemia, but they predispose to seizures in cases of elevated blood glucose. They include acute focal or pre-existing lesions, such as cortical dysplasia, heterotopia, strokes, and encephalomalacia from previous insults [11].

Hyperglycemia-induced seizures tend to be refractory to anti-epileptic medications, and some anti-epileptic drugs like phenytoin may exacerbate and worsen seizures as they inhibit insulin secretions [6]. Hence, early recognition of the presence of hyperglycemia as the main driving cause of seizures is important in order to start the right management [12]. Lowering blood glucose with insulin and hydration is the mainstay of treatment. In a study of 13 cases of patients with non-ketotic hyperglycemia, the combination of anti-epileptics and insulin therapy with proper hydration achieved a seizure-free state in all of the patients, and then anti-epileptics were gradually stopped [8]. 

We added some of the reported cases of non-ketotic hyperglycemia presenting as seizure in Table 1. Most of the cases presented as focal motor seizure, some of them were in focal status epilepticus, a large number of the patients responded well to intravenous hydration and insulin therapy, and some of them needed anti-epileptic drugs for better seizure control.

**Table 1:** Selected cases of non-ketotic hyperglycemia presenting as seizure.

Age, Reference	Sex	Diabetes status	HbA1c level	Type of seizure	MRI result	EEG	Management	Notes
70, [1]	F	Newly diagnosed	11.2%	Focal motor, left side	Hyposignal ADC and FLAIR in the occipital region	-	-	
73, [1]	M	Type 2 DM	11.6%	4 GTCS	Hyposignal ADC and FLAIR in the right frontal region	-	-	
75, [2]	F	Type 2 DM	15.4%	Focal status epilepticus, motor, left side	Right subcortical frontal lesion, T1 isointense and T2 hyperintense with post-contrast enhancement, suggestive of low-grade glioma	Normal	Hydration Insulin therapy	Although there was a space-occupying lesion, all the episodes of the experienced focal seizures were during severe hyperglycemia, when blood glucose was above 500 mg/dl
21, [3]	F	DM type 2	12.1%	Focal status epilepticus, motor, right side	T2 FLAIR subcortical hypointensity and cortical hyperintensity	Generalized spikes and polyspikes	Carbamazepine, clobazam, insulin therapy	
37, [5]	M	Newly diagnosed	-	Focal motor, right side	-	-	Carbamazepine, hydration insulin infusion	CT head is normal
62, [5]	F	Newly diagnosed	-	Focal motor, left side	-	-	Phenytoin, glibenclamide	CT showed right perisylvian atrophy
85, [7]	M	Newly diagnosed	10.2%	Focal motor, left side	No new vascular insult, chronic small vessel disease	-	Hydration insulin therapy	
83, [11]	M	DM type 2	-	Left focal motor and occipital seizures	Not done due to the presence of a pacemaker	Electrographic seizures from the left occipital region	Hydration insulin therapy	CT head is normal
57, [12]	M	Newly diagnosed	-	Focal status epilepticus, motor, right side	No abnormalities seen	Irregular, sharp, and slow waves on the right temporal region with contralateral propagation	Carbamazepine valproic acid, hydration insulin therapy	Initially was treated for his seizures only, as he wasn’t known to have DM, on discovery he was treated appropriately as he was found to have NKH
75	F	DM type 2	11.2%	Focal status epilepticus, motor, left side	Small vessel disease	Normal	Levetiracetam, hydration insulin therapy	Our case
ADC: apparent diffusion coefficient, DM: diabetes mellitus, F: female, FLAIR: fluid-attenuated inversion recovery, GTCS: generalized tonic-clonic seizure, M: male, NKH: non-ketotic hyperglycemia								

## Conclusions

Non-ketotic hyperglycemia-induced seizures present mainly as focal motor seizures. Patients with uncontrolled diabetes who are above the age of 50 are at risk; in our case, our patient was 75 years old and had uncontrolled diabetes. Management, in general, is challenging as it depends on controlling blood glucose. Hence, the time required is variable. Our patient, who presented with focal status epilepticus lasting more than two hours, was given lorazepam and a loading dose of levetiracetam. She was on a maintenance dose of the same anti-epileptic while the glucose was being corrected as per the hospital’s protocol. During her hospital stay, it was tapered down slowly and eventually stopped prior to discharge. There was no recurrence of seizures after admission.

## References

[1] Baltyde D, De Toffol B, Nacher M, Sabbah N (2022). Epileptic seizures during non-ketotic hyperglycemia (NKH) in French Guiana: a retrospective study. Front Endocrinol (Lausanne).

[2] Chatterjee S, Ghosh R, Ojha UK, Diksha Diksha, Biswas P, Benito-León J, Dubey S (2022). Recurrent facial focal seizures with chronic striatopathy and caudate atrophy—a double whammy in an elderly woman with diabetes mellitus. Neurohospitalist.

[3] Gorijala VK, Shaik L, Kowtha P, Kaur P, Nagarjunakonda VS (2020). A case report of nonketotic hyperglycemic seizures: a diagnostic dilemma. Cureus.

[4] Peddawad D (2022). Epileptic manifestations, pathophysiology, and imaging characteristics of non-ketotic hyperglycaemia: a review of the literature and a report of two cases with irreversible cortical vision loss. J Int Med Res.

[5] Hennis A, Corbin D, Fraser H (1992). Focal seizures and non-ketotic hyperglycaemia. J Neurol Neurosurg Psychiatry.

[6] Younes S, Cherif Y, Aissi M (2014). Seizures and movement disorders induced by hyperglycemia without ketosis in elderly. Iran J Neurol.

[7] Odak M, Douedi S, Upadhyaya V, Fadhel M, Cosentino J (2020). Focal neurological seizure due to hyperglycemic hyperosmolar non-ketotic syndrome in undiagnosed diabetes mellitus. Cureus.

[8] Wang X (2017). Nonketotic hyperglycemia-related epileptic seizures. Chin Neurosurg J.

[9] Hiremath SB, Gautam AA, George PJ, Thomas A, Thomas R, Benjamin G (2019). Hyperglycemia-induced seizures-understanding the clinico-radiological association. Indian J Radiol Imaging.

[10] De Martino SR, Toni F, Spinardi L, Cirillo L (2020). Magnetic resonance imaging findings in patients with non-ketotic hyperglycaemia and focal seizures. Neuroradiol J.

[11] Moien-Afshari F, Téllez-Zenteno JF (2009). Occipital seizures induced by hyperglycemia: a case report and review of literature. Seizure.

[12] Cokar O, Aydin B, Ozer F (2004). Non-ketotic hyperglycaemia presenting as epilepsia partialis continua. Seizure.

